# (*E*)-2-[(4-Iodo­phen­yl)imino­meth­yl]-6-methyl­phenol

**DOI:** 10.1107/S160053681001826X

**Published:** 2010-06-05

**Authors:** Gonca Özdemir Tarı, Umit Ceylan, Mustafa Macit, Şamil Isık

**Affiliations:** a Department of Physics, Faculty of Arts & Science, Ondokuz Mayıs University, TR-55139 Kurupelit-Samsun, Turkey; b Department of Chemistry, Faculty of Arts & Science, Ondokuz Mayıs University, 55139 Samsun, Turkey

## Abstract

The title compound, C_14_H_12_INO, adopts the phenol–imine tautomeric form. The dihedral angle between the aromatic rings is 20.6 (3)°. The mol­ecular conformation is stabilized by an intra­molecular O—H⋯N hydrogen bond while in the crystal, weak inter­molecular C—H⋯O hydrogen bonds link the mol­ecules into a zigzag chain parallel to the *b* axis.

## Related literature

For background to the properties and uses of Schiff bases, see: Barton & Ollis (1979 ▶); Layer (1963 ▶); Ingold (1969 ▶); Cohen *et al.* (1964 ▶); Taggi *et al.* (2002 ▶). For hydrogen-bond motifs, see: Bernstein *et al.* (1995 ▶). For comparative bond lengths, see: Şahin *et al.* (2009 ▶). For related structures, see: Özdemir *et al.* (2010 ▶); Tanak *et al.* (2009 ▶).
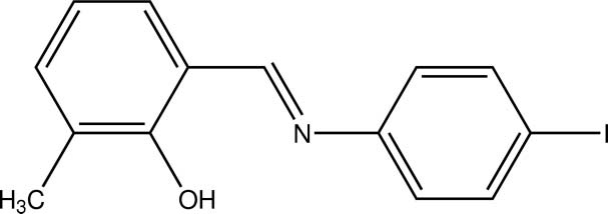


## Experimental

### 

#### Crystal data

C_14_H_12_INO
*M* *_r_* = 337.15
Orthorhombic, 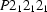

*a* = 4.6773 (4) Å
*b* = 11.6092 (12) Å
*c* = 23.6751 (4) Å
*V* = 1285.55 (17) Å^3^
*Z* = 4
Mo *K*α radiation
μ = 2.47 mm^−1^
*T* = 293 K
0.48 × 0.24 × 0.09 mm

#### Data collection

Stoe IPDS II diffractometer
Absorption correction: numerical (*X-AREA*; Stoe & Cie, 2002 ▶) *T*_min_ = 0.520, *T*_max_ = 0.769
7548 measured reflections
2267 independent reflections
1541 reflections with *I* > 2σ(*I*)
*R*_int_ = 0.086

#### Refinement

*R*[*F* ^2^ > 2σ(*F* ^2^)] = 0.037
*wR*(*F* ^2^) = 0.078
*S* = 0.86
2267 reflections
156 parameters
H-atom parameters constrained
Δρ_max_ = 0.65 e Å^−3^
Δρ_min_ = −0.29 e Å^−3^
Absolute structure: Flack (1983 ▶), 901 Friedel pairs
Flack parameter: 0.10 (5)

### 

Data collection: *X-AREA* (Stoe & Cie, 2002 ▶); cell refinement: *X-AREA*; data reduction: *X-AREA*; program(s) used to solve structure: *SHELXS97* (Sheldrick, 2008 ▶); program(s) used to refine structure: *SHELXL97* (Sheldrick, 2008 ▶); molecular graphics: *ORTEPIII* (Burnett & Johnson, 1996 ▶), *ORTEP-3 for Windows* (Farrugia, 1997 ▶) and *PLATON* (Spek, 2009 ▶); software used to prepare material for publication: *SHELXL97*.

## Supplementary Material

Crystal structure: contains datablocks I, global. DOI: 10.1107/S160053681001826X/dn2563sup1.cif

Structure factors: contains datablocks I. DOI: 10.1107/S160053681001826X/dn2563Isup2.hkl

Additional supplementary materials:  crystallographic information; 3D view; checkCIF report

## Figures and Tables

**Table 1:** Hydrogen-bond geometry (Å, °)

*D*—H⋯*A*	*D*—H	H⋯*A*	*D*⋯*A*	*D*—H⋯*A*
O1—H1⋯N1	0.82	1.86	2.591 (8)	147
C13—H13⋯O1^i^	0.93	2.51	3.348 (8)	150

Symmetry code: (i) 
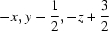
.

**Fig. 1.**
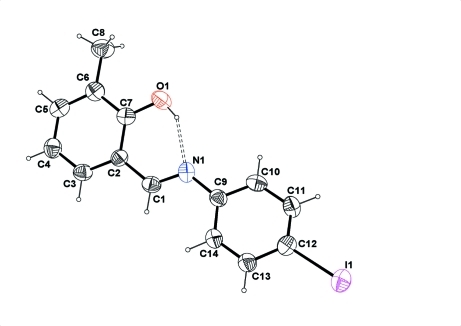
The molecular structure of the title compound, showing the atom-numbering scheme and 30% probability displacement ellipsoids. H atoms are represented as small spheres of arbitrary radii. H bond is shown as dashed lines.

**Fig. 2.**
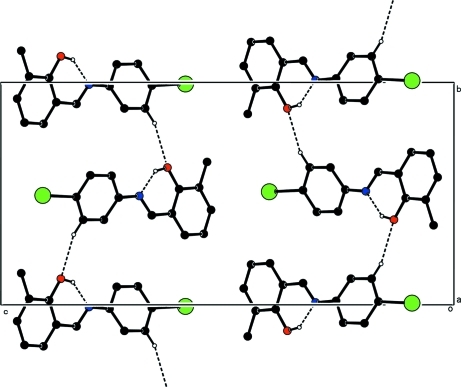
Partial packing view showing the formation of zig-zag chain parallel to the b axis. H atoms not involved in hydrogen bondings have been omitted for clarity. C-H···O hydrogen bonds are represented as dashed lines

## supplementary crystallographic information

### Comment

Schiff bases are used as starting materials in the synthesis of important drugs, such as antibiotics and antiallergic, antiphlogistic, and antitumor substances (Barton *et al.*, 1979; Layer, 1963; Ingold 1969). On the industrial scale, they have a wide range of applications, such as dyes and pigments (Taggi *et al.*, 2002). There are two characteristic properties of Schiff bases, viz. Photochromism and thermochromism (Cohen *et al.*, 1964). In general, Schiff bases display two possible tautomeric forms, the phenol-imine (OH) and the keto-amine (NH) forms. Depending on the tautomers, two types of intramolecular hydrogen bonds are observed in Schiff bases: O—H···N in phenol-imine (Şahin *et al.*, 2009) and N—H···O in keto-amine tautomers (Tanak *et al.*, 2009). Another form of the Schiff base compounds is also known as zwitterion having an ionic intramolecular hydrogen bond (N^+^—H···O^-^) and this form is rarely seen in the solid state (Özdemir *et al.*, 2010).

The molecular structure of the title compound, C_14_H_17_O_1_N_1_I_1, _shows that the molecule exists in the phenol-imine form (Fig. 1). The C1=N1 [1.269 (8) Å] and C9=N1 [1.397 (7) Å] bond distances are of double-bond character, whereas, C7—O1 [1.332 (8) Å] distance is single bond. These distances are similar to that reported in the literature [1.277 (3) Å] and [1.402 (3) Å] for C=N and [1.347 (3) Å] for C—O respectively (Şahin *et al.*, 2009).

The molecule of title compound is non-planar (Fig. 1), the two phenyl rings are twisted by a dihedral angle of 20.6 (3)°. This conformation is stabilized by intramolecular N-H···O hydrogen bond (Table 1, Fig. 1) forming S(6) ring (Bernstein *et al.*, 1995). weak intermolecular C-H···O hydrogen bonds link the molecules forming a zig-zag chain parallel to the b axis (Table 1, Fig. 2). The I atom is slightly out of the C9-C14 ring by 0.18 (1)Å.

### Experimental

The compound (*E*)-2-[(4-Iodophenylimino)methyl]-6-methylphenol was prepared by reflux a mixture of a solution containing 3-methylsalicylaldehyde (0.1 ml 0.82 mmol) in 20 ml e thanol and solution containing 4-Iodoaniline (0.179 g 0.82 mmol) in 20 ml e thanol.The reaction mixture was stirred for 1 hunder reflux. The crystals of (*E*)-2-[(4-Iodophenylimino)methyl]-6-methylphenol suitable for x-ray analysis were obtained from ethylalcohol by slow evaporation (yield 51%; m.p.350-353 K).

### Refinement

The position of the H1 atom was obtained from a difference map of the electron density in the unit-cell and was refined freely. Other H atoms were positioned geometrically and treated using a riding model, fixing the bond lengths at 0.93 Å for aromatic CH and at 0.96 Å for CH_3_. The displacement parameters of the H atoms were constrained as U_iso_(H)= 1.2U_eq_(1.5U_eq_ for methyl) of the parent atom.

### Crystal data

**Table:**

C_14_H_12_INO	*F*(000) = 656
*M**_r_* = 337.15	*D*_x_ = 1.742 Mg m^−^^3^
Orthorhombic, *P*2_1_2_1_2_1_	Mo *K*α radiation, λ = 0.71073 Å
Hall symbol: P 2ac 2ab	Cell parameters from 7912 reflections
*a* = 4.6773 (4) Å	θ = 1.7–27.8°
*b* = 11.6092 (12) Å	µ = 2.47 mm^−^^1^
*c* = 23.6751 (4) Å	*T* = 293 K
*V* = 1285.55 (17) Å^3^	Prism, yellow
*Z* = 4	0.48 × 0.24 × 0.09 mm

### Data collection

**Table:**

Stoe IPDS II diffractometer	2267 independent reflections
Radiation source: fine-focus sealed tube	1541 reflections with *I* > 2σ(*I*)
graphite	*R*_int_ = 0.086
Detector resolution: 6.67 pixels mm^-1^	θ_max_ = 25.0°, θ_min_ = 1.7°
rotation method scans	*h* = −5→5
Absorption correction: numerical (*X-AREA*; Stoe & Cie, 2002)	*k* = −13→13
*T*_min_ = 0.520, *T*_max_ = 0.769	*l* = −28→28
7548 measured reflections	

### Refinement

**Table:**

Refinement on *F*^2^	Secondary atom site location: difference Fourier map
Least-squares matrix: full	Hydrogen site location: inferred from neighbouring sites
*R*[*F*^2^ > 2σ(*F*^2^)] = 0.037	H-atom parameters constrained
*wR*(*F*^2^) = 0.078	*w* = 1/[σ^2^(*F*_o_^2^) + (0.0321*P*)^2^] where *P* = (*F*_o_^2^ + 2*F*_c_^2^)/3
*S* = 0.86	(Δ/σ)_max_ = 0.001
2267 reflections	Δρ_max_ = 0.65 e Å^−^^3^
156 parameters	Δρ_min_ = −0.29 e Å^−^^3^
0 restraints	Absolute structure: Flack (1983), 901 Friedel pairs
Primary atom site location: structure-invariant direct methods	Flack parameter: 0.10 (5)

### Special details

**Table:**

Geometry. All esds (except the esd in the dihedral angle between two l.s. planes) are estimated using the full covariance matrix. The cell esds are taken into account individually in the estimation of esds in distances, angles and torsion angles; correlations between esds in cell parameters are only used when they are defined by crystal symmetry. An approximate (isotropic) treatment of cell esds is used for estimating esds involving l.s. planes.
Refinement. Refinement of *F*^2^ against ALL reflections. The weighted *R*-factor wR and goodness of fit *S* are based on *F*^2^, conventional *R*-factors *R* are based on *F*, with *F* set to zero for negative *F*^2^. The threshold expression of *F*^2^ > σ(*F*^2^) is used only for calculating *R*-factors(gt) etc. and is not relevant to the choice of reflections for refinement. *R*-factors based on *F*^2^ are statistically about twice as large as those based on *F*, and *R*- factors based on ALL data will be even larger.

### Fractional atomic coordinates and isotropic or equivalent isotropic displacement parameters (Å^2^)

**Table:**

	*x*	*y*	*z*	*U*_iso_*/*U*_eq_	
I1	0.81565 (9)	0.49212 (4)	0.907466 (15)	0.07878 (18)	
O1	−0.3098 (17)	0.6170 (3)	0.6324 (2)	0.0759 (15)	
H1	−0.2028	0.6014	0.6588	0.114*	
N1	0.0187 (10)	0.4895 (5)	0.69451 (16)	0.0621 (12)	
C1	−0.0685 (14)	0.4026 (6)	0.6669 (2)	0.0596 (16)	
H15	0.0064	0.3306	0.6757	0.071*	
C2	−0.2788 (18)	0.4106 (5)	0.6224 (3)	0.0553 (19)	
C3	−0.3704 (13)	0.3134 (5)	0.5941 (3)	0.0639 (16)	
H3	−0.2933	0.2423	0.6037	0.077*	
C4	−0.5711 (17)	0.3190 (6)	0.5522 (3)	0.071 (2)	
H4	−0.6369	0.2523	0.5348	0.085*	
C5	−0.6755 (18)	0.4266 (6)	0.5361 (3)	0.0680 (18)	
H5	−0.8058	0.4307	0.5065	0.082*	
C6	−0.5925 (14)	0.5272 (6)	0.5624 (3)	0.0660 (19)	
C7	−0.3895 (13)	0.5201 (5)	0.6065 (2)	0.0568 (15)	
C8	−0.699 (2)	0.6422 (6)	0.5449 (3)	0.091 (3)	
H8A	−0.8417	0.6332	0.5161	0.137*	
H8B	−0.5434	0.6872	0.5305	0.137*	
H8C	−0.7818	0.6806	0.5769	0.137*	
C9	0.2092 (13)	0.4822 (5)	0.7398 (2)	0.0582 (13)	
C10	0.3469 (18)	0.5806 (5)	0.7557 (3)	0.068 (2)	
H10	0.3162	0.6479	0.7353	0.082*	
C11	0.5354 (18)	0.5827 (6)	0.8024 (3)	0.069 (2)	
H11	0.6340	0.6496	0.8118	0.083*	
C12	0.5693 (13)	0.4850 (7)	0.8333 (2)	0.0636 (15)	
C13	0.4354 (17)	0.3826 (6)	0.8184 (3)	0.0658 (19)	
H13	0.4652	0.3158	0.8392	0.079*	
C14	0.2599 (16)	0.3824 (5)	0.7726 (3)	0.068 (2)	
H14	0.1697	0.3141	0.7625	0.081*	

### Atomic displacement parameters (Å^2^)

**Table:**

	*U*^11^	*U*^22^	*U*^33^	*U*^12^	*U*^13^	*U*^23^
I1	0.0755 (3)	0.0930 (3)	0.0679 (2)	−0.0021 (4)	−0.0059 (2)	−0.0085 (3)
O1	0.099 (5)	0.053 (2)	0.076 (3)	−0.001 (3)	0.001 (3)	−0.001 (2)
N1	0.060 (3)	0.068 (3)	0.058 (2)	−0.011 (4)	0.002 (2)	−0.007 (3)
C1	0.058 (4)	0.056 (4)	0.064 (4)	0.008 (3)	0.009 (3)	0.002 (3)
C2	0.050 (5)	0.060 (4)	0.057 (3)	0.004 (3)	0.001 (3)	−0.001 (3)
C3	0.068 (5)	0.060 (3)	0.064 (3)	0.012 (3)	0.003 (4)	−0.003 (3)
C4	0.071 (5)	0.076 (5)	0.067 (4)	−0.004 (4)	0.000 (4)	−0.010 (3)
C5	0.060 (4)	0.086 (5)	0.058 (4)	0.005 (5)	0.006 (4)	0.010 (3)
C6	0.066 (4)	0.072 (5)	0.060 (3)	−0.007 (4)	0.011 (3)	0.012 (3)
C7	0.059 (4)	0.061 (4)	0.051 (3)	0.002 (4)	0.012 (2)	0.007 (3)
C8	0.109 (9)	0.080 (5)	0.085 (5)	0.012 (6)	−0.006 (5)	0.016 (4)
C9	0.058 (3)	0.052 (3)	0.066 (3)	0.003 (4)	0.003 (3)	−0.006 (3)
C10	0.078 (6)	0.054 (4)	0.073 (4)	0.005 (4)	0.007 (4)	0.014 (3)
C11	0.078 (5)	0.064 (4)	0.067 (4)	−0.017 (4)	0.001 (4)	−0.005 (3)
C12	0.063 (3)	0.076 (5)	0.052 (3)	0.000 (4)	0.004 (2)	0.001 (4)
C13	0.075 (5)	0.060 (4)	0.063 (4)	−0.001 (4)	−0.004 (4)	−0.002 (3)
C14	0.074 (7)	0.058 (4)	0.071 (4)	−0.006 (4)	−0.010 (4)	−0.007 (3)

### Geometric parameters (Å, °)

**Table:**

I1—C12	2.102 (5)	C6—C7	1.415 (8)
O1—C7	1.334 (7)	C6—C8	1.484 (9)
O1—H1	0.8200	C8—H8A	0.9600
N1—C1	1.269 (8)	C8—H8B	0.9600
N1—C9	1.397 (7)	C8—H8C	0.9600
C1—C2	1.444 (10)	C9—C10	1.364 (9)
C1—H15	0.9300	C9—C14	1.414 (9)
C2—C3	1.381 (8)	C10—C11	1.415 (10)
C2—C7	1.423 (9)	C10—H10	0.9300
C3—C4	1.367 (9)	C11—C12	1.358 (9)
C3—H3	0.9300	C11—H11	0.9300
C4—C5	1.394 (10)	C12—C13	1.389 (10)
C4—H4	0.9300	C13—C14	1.359 (9)
C5—C6	1.379 (9)	C13—H13	0.9300
C5—H5	0.9300	C14—H14	0.9300
			
C7—O1—H1	109.5	C6—C8—H8B	109.5
C1—N1—C9	123.5 (6)	H8A—C8—H8B	109.5
N1—C1—C2	122.9 (6)	C6—C8—H8C	109.5
N1—C1—H15	118.5	H8A—C8—H8C	109.5
C2—C1—H15	118.5	H8B—C8—H8C	109.5
C3—C2—C7	119.2 (6)	C10—C9—N1	117.5 (6)
C3—C2—C1	120.9 (6)	C10—C9—C14	117.1 (5)
C7—C2—C1	119.9 (6)	N1—C9—C14	125.3 (6)
C4—C3—C2	121.8 (6)	C9—C10—C11	121.6 (6)
C4—C3—H3	119.1	C9—C10—H10	119.2
C2—C3—H3	119.1	C11—C10—H10	119.2
C3—C4—C5	118.8 (6)	C12—C11—C10	118.6 (6)
C3—C4—H4	120.6	C12—C11—H11	120.7
C5—C4—H4	120.6	C10—C11—H11	120.7
C6—C5—C4	122.4 (6)	C11—C12—C13	121.7 (5)
C6—C5—H5	118.8	C11—C12—I1	118.7 (5)
C4—C5—H5	118.8	C13—C12—I1	119.5 (5)
C5—C6—C7	118.3 (6)	C14—C13—C12	118.4 (6)
C5—C6—C8	122.8 (6)	C14—C13—H13	120.8
C7—C6—C8	118.9 (7)	C12—C13—H13	120.8
O1—C7—C6	118.6 (6)	C13—C14—C9	122.5 (6)
O1—C7—C2	122.0 (5)	C13—C14—H14	118.7
C6—C7—C2	119.4 (6)	C9—C14—H14	118.7
C6—C8—H8A	109.5		
			
C9—N1—C1—C2	176.1 (5)	C3—C2—C7—C6	0.5 (9)
N1—C1—C2—C3	−179.1 (6)	C1—C2—C7—C6	179.1 (5)
N1—C1—C2—C7	2.3 (9)	C1—N1—C9—C10	162.2 (6)
C7—C2—C3—C4	−2.0 (10)	C1—N1—C9—C14	−21.4 (9)
C1—C2—C3—C4	179.4 (6)	N1—C9—C10—C11	177.6 (6)
C2—C3—C4—C5	3.1 (10)	C14—C9—C10—C11	0.8 (10)
C3—C4—C5—C6	−2.8 (11)	C9—C10—C11—C12	−2.7 (11)
C4—C5—C6—C7	1.3 (10)	C10—C11—C12—C13	3.3 (11)
C4—C5—C6—C8	179.1 (7)	C10—C11—C12—I1	−173.8 (5)
C5—C6—C7—O1	−179.6 (6)	C11—C12—C13—C14	−2.0 (11)
C8—C6—C7—O1	2.6 (9)	I1—C12—C13—C14	175.1 (5)
C5—C6—C7—C2	−0.2 (9)	C12—C13—C14—C9	0.1 (11)
C8—C6—C7—C2	−178.0 (6)	C10—C9—C14—C13	0.5 (10)
C3—C2—C7—O1	179.9 (7)	N1—C9—C14—C13	−176.0 (6)
C1—C2—C7—O1	−1.5 (9)	C2—C1—N1—C9	176.1 (5)

### Hydrogen-bond geometry (Å, °)

**Table:**

*D*—H···*A*	*D*—H	H···*A*	*D*···*A*	*D*—H···*A*
O1—H1···N1	0.82	1.86	2.591 (8)	147
C13—H13···O1^i^	0.93	2.51	3.348 (8)	150

Symmetry codes: (i) −*x*, *y*−1/2, −*z*+3/2.
